# C5a and C5aR are elevated in joints of rheumatoid and psoriatic arthritis patients, and C5aR blockade attenuates leukocyte migration to synovial fluid

**DOI:** 10.1371/journal.pone.0189017

**Published:** 2017-12-08

**Authors:** Lars Hornum, Anker Jon Hansen, Ditte Tornehave, Marianne Scheel Fjording, Paula Colmenero, Inger Falbe Wätjen, Niels Henrik Søe Nielsen, Henning Bliddal, Else Marie Bartels

**Affiliations:** 1 Novo Nordisk A/S, Måløv, Denmark; 2 Radcliffe Department of Medicine, University of Oxford, Oxford, United Kingdom; 3 The Parker Institute, Copenhagen University Hospital, Bispebjerg and Frederiksberg, Frederiksberg, Denmark; 4 Hand Section, Department of Orthopaedics, Herlev and Gentofte Hospital, Gentofte, Denmark; 5 Faculty of Health and Medical Sciences, University of Copenhagen, Copenhagen, Denmark; University of Leicester, UNITED KINGDOM

## Abstract

Complement activation correlates to rheumatoid arthritis disease activity, and increased amounts of the complement split product C5a is observed in synovial fluids from rheumatoid arthritis patients. Blockade of C5a or its receptor (C5aR) is efficacious in several arthritis models. The aim of this study was to investigate the role of C5a and C5aR in human rheumatoid arthritis and psoriatic arthritis–both with respect to expression and function. Synovial fluid, blood and synovial samples were obtained from rheumatoid arthritis, psoriatic arthritis and osteoarthritis patients as a less inflammatory arthritis type, and blood from healthy subjects. Cells infiltrating synovial tissue were analysed by immunohistochemistry and flow cytometry. SF and blood were analysed for biomarkers by flow cytometry or ELISA. The effect of a blocking anti-human C5aR mAb on leukocyte migration was determined using a Boyden chamber. Appropriate statistical tests were applied for comparisons. C5aR^+^ cells were detected in most rheumatoid arthritis, in all psoriatic arthritis, but not in non-inflammatory control synovia. C5aR^+^ cells were primarily neutrophils and macrophages. C5aR^+^ macrophages were mainly found in lymphoid aggregates in close contact with T cells. C5a levels were increased in both rheumatoid arthritis and psoriatic arthritis synovial fluid compared to osteoarthritis, and in blood from rheumatoid arthritis compared to healthy subjects. Neutrophil and monocyte migration to rheumatoid arthritis synovial fluid was significantly inhibited by anti-C5aR. The data support that the C5a-C5aR axis may be driving the infiltration of inflammatory cells into the synovial fluid and synovium in both rheumatoid and psoriatic arthritis, and suggest that C5a or C5aR may be a promising treatment target in both diseases.

## Introduction

The complement system plays a central role in the immune system by constituting a non-cellular system for protection against pathogens via a protease cascade leading to the activation of several effector molecules downstream of three activation pathways (classical, lectin and alternative). Effector molecules include C3b leading to opzonization, the membrane attack complex directly leading to cell lysis, and the formation of anaphylatoxins (C3a and C5a) with chemotactic and other pro-inflammatory properties [1]. C5a exerts its function on cells expressing the C5aR receptor. An alternative non-signalling receptor is identified, but the function of this receptor is disputed, but data indicates that C5L2 functions as a intracellular inhibitor of C5a-induced C5aR signalling [2, 3].

The complement system is suggested to play a major role in rheumatoid arthritis (RA) pathogenesis, where complement activation products are found to be increased in synovial fluid (SF) to higher levels than in the matching plasma [4]. Moreover, the level of complement activation correlates with disease activity [5–7]. Complement polymorphism is shown as a likely player when increasing the concentration of active C5a in RA joints [8].

One of the major effector molecules in the complement system is the split product C5a of complement C5. This highly proinflammatory molecule acts on C5a-receptor (C5aR) positive cells, which include granulocytes, monocytes, subsets of macrophages, dendritic cells and mast cells, while expression on T cells is debated [9, 10]. C5a is a potent chemoattractant, but is also activating C5aR-positive cells: It induces oxidative bursts and release of effector molecules from neutrophils, and cytokines from monocytes and macrophages. A number of studies demonstrate, furthermore, that C5a enhances T-cell activation, most likely via action on C5aR-positive cells [11], and C5a is shown to be upregulated in RA SF [12, 13]. In contrast to RA, no data exist on the role of complement in the pathogenesis of psoriatic arthritis (PsA) [14].

Complement is necessary in arthritis development in the collagen-induced arthritis (CIA) model [15], and an active role of C5a has also been implicated in other arthritis models: C5aR deficiency protects against arthritis in collagen antibody-induced (CAIA), the chronic autoimmune SKG, and the K/BxN serum transfer models [16–19], while anti-C5a or–C5aR blockade is efficacious in collagen-induced (CIA) and rat antigen-induced arthritis [20, 21]. We specifically demonstrated a therapeutic effect of an anti-C5aR mAb in the CIA model, where C5aR blockade inhibited infiltration of monocytes and neutrophils into the paw, and decreased levels of several pro-inflammatory cytokines [22].

In RA monocytes migrate into the synovial tissue and differentiate into macrophages [23]. Number of synovial macrophages is correlated to level of local disease activity and joint destruction [24, 25], and synovial macrophages have been identified as the best clinical response marker for glucocorticosteroids, methotrexate, leflunomide and infliximab [26, 27]. Furthermore, the beneficial effect of antibodies targeting TNFα, IL-1, and IL-6 may support the role of macrophages in RA pathogenesis, since macrophages produce high amounts of these cytokines. Other cells, including neutrophils, also express these cytokines [28]. Neutrophils are the most abundant cell type in RA SF [29], and when activated produce effector molecules and process endocytosed material [30–35]. Neutrophils are also found in the synovium, mostly in the pannus/articular junction, where they are associated with complement deposition and proteoglycan degradation [36–40]. Cases where increase in neutrophil counts upon G-CSF treatment of neutropenia in Felty’s syndrome is, furthermore, associated with RA flare [41–43]. Finally, in RA glucocorticoids, leflunomide, methotrexate and anti-TNFα mAbs inhibit neutrophil influx into inflamed joints [44–48]. Several lines of evidence therefore support a pathogenic role of neutrophils in RA.

Based on the hypothesis that the C5a-C5aR axis is playing a major role in initiating and/or maintaining inflammatory joint diseases, our aim was to investigate C5a and C5aR presence in blood and SF from RA and PsA. Our approach was first to perform immunohistochemical analyses of synovia from one patient cohort to identify infiltrating C5aR-positive cells. Then we measured the levels of C5a in blood and SF in another cohort to see whether these correlate to the level of systemic and local inflammation, and to the number of neutrophils recruited to the SF. Finally, we performed functional assays analysing the impact of C5aR blockade on neutrophil and monocyte migration towards patient SF.

Our results indicate that the C5a-C5aR axis is important in the onset of inflammation and driving the influx of immune cells into the synovium in RA and PsA.

## Materials and methods

The protocol for this study can be accessed from protocols.io, DOI: 10.17504/protocols.io.kmxcu7n.

### Immunohistochemistry

#### Human material

Human tissue samples from RA, PsA and OA, as well as from non-inflammatory controls, used in the present study are listed in Table 1.

**Table 1:** Human tissues used in the present study.

Non-inflammatory control^1^	Number of samples	Provider
Synovium^2^^,^^3^	7	Biochain Institute Inc./BioCat GmbH, Heidelberg, Germany Cambridge BioScience (Cambridge, UK)
**Inflamed Synovial Tissue**		
Rheumatoid arthritis–Synovial biopsies obtained from patients undergoing joint replacements^2^^,^^3^^,^^4^	35	Biochain Institute Inc./BioCat GmbH, Heidelberg, Germany Cambridge BioScience (Cambridge, UK)
Rheumatoid arthritis–Synovial biopsies obtained from patients undergoing synovectomy^5^^,^^6^	8	Parker Institute Copenhagen, DK
Osteoarthritis—Synovial biopsies obtained from patients undergoing joint replacements^2^^,^^3^^,^^4^^,^^7^	27	Biochain Institute Inc./BioCat GmbH, Heidelberg, Germany Cambridge BioScience (Cambridge, UK)
Psoriatic arthritis—Synovial biopsies obtained from patients undergoing joint replacements^2^^,^^3^^,^^4^^,^^7^	10	Biochain Institute Inc./BioCat GmbH, Heidelberg, Germany Cambridge BioScience (Cambridge, UK)

^1^Histopathologically classified as being within normal limits.

^2^Obtained post-operational.

^3^Tissue microarrays (TMA) containing cores of synovial biopsies. Biopsies obtained from

^4^Knee

^5^Finger

^6^Wrist, and

^7^Hip joints.

#### Ethics

All human materials were obtained with written informed consent from the donors and approval from local ethical committees (H-4-2009 DK; BioCat Ge; Cambridge BioSciences, Supplier information: Tissue Supply Network (http://www.bioscience.co.uk)).

#### Sectioning

Fixed paraffin embedded cell and tissue sections were cut at a nominal thickness of 3 μm, mounted on Superfrost^®^Plus microscope slides, and stored at -20°C until further processing.

#### Antibodies

Antibodies and conjugated detection kits used for immunohistochemical studies are listed in Table A1 in S1 Text.

#### Immunohistochemical methods

Immunohistochemical (IHC) staining for C5aR and double-immunofluorescence staining for characterization of C5aR^+^ cells were carried out and is described in S1 Text.

#### Microscopic analyses

Sections were evaluated by virtual microscopy using either a Hamamatsu NanoZoomer (Hamamatsu Denmark, Ballerup, Denmark), or a FV10i Laser scanning microscope (Olympus Denmark A/S; Ballerup, Denmark), and an Olympus BX51 reflected fluorescence system microscope equipped with selective AMCA, FITC and Texas-Red filters, and a DP70 digital camera (Olympus Denmark A/S; Ballerup, Denmark).

### Flow cytometry analysis of RA synovial tissue cells

#### Tissue and ethics

RA synovial membranes were obtained from patients undergoing joint replacement surgery or synovectomy at a London hospital and fulfilling the American College of Rheumatology (ACR) criteria for RA [49], and who had given written informed consent to participation in this study. The local ethics committee approved the study (London Riverside Research Ethics Committee, 1752). The tissues came from 1 hip joint, 3 knee joints, 2 elbow joints, 1 wrist joint and 2 from hand joints.

#### Cell preparation

Single-cell suspensions of RA synovial tissue biopsies were prepared as described in S2 Text.

### Study of matching samples of synovial fluid and blood

#### Patients and ethics

For assessment of paired blood and SF samples, a total of 30 patients with active RA, 10 patients with active PsA, and 19 patients with active OA were recruited from the day clinic at the Parker Institute, Copenhagen University Hospital, Bispebjerg and Frederiksberg. OA was included, since it was considered to present swollen knees which were less inflammatory than RA and PsA, and non-inflammatory controls were not available due to ethical issues. A healthy control group (HC), 30 age- and sex-matched subjects, were recruited via the Copenhagen City Heart Study (https://www.herlevhospital.dk/afdelinger-og-klinikker/klinisk-biokemisk-afdeling/forskning/Sider/Herlev-oesterbroundersoegelsen.aspx), or via advertising, for blood sampling. The study was approved by the Capital Region of Denmark’s ethical committee (No. H-4-2009-117), and was carried out according to the Helsinki Declaration (http://www.wma.net/en/30publications/10policies/b3/index.html).

For patients, inclusion criteria were: Written informed consent given; A diagnosis of RA according to the ACR1987 criteria [49], or PsA defined according to ‘Classification criteria for Psoriatic arthritis’ (CASPAR) [47], or OA according to the ACR-criteria [50]; Active arthritis with joint swelling and tenderness.

Exclusion criteria were: An alcohol consumption of more than an amount equivalent to 1 unit of alcohol 12 hours or less prior to sampling; Active medical treatment for other chronic diseases than the arthritic condition; Prior to study enrolment in active treatment with any of the following medications: Glucocorticoid unless given in stable doses equivalent to ≤ 10 mg of prednisone orally /day the preceding month, or intra-articular, intra-muscular or intra-venous corticosteroids within the last month, or Cyclosporine, Azathioprine, Penicillamine, Etanercept, Infliximab, Abatacept, Efalizumab, Alefacept, Adalimumab, intravenous immunogammaglobulins (IVIG), Rituximab, or Gold therapy. During the course of the disease, it was acceptable that the patients previously had been in active treatment with any of the above listed medications. In such cases, the treatment must have been discontinued at least 1 month prior to enrolment into the study.

For HC, inclusion criteria were: Written informed consent given; Not suffering from any disease, acute or chronic; No regular intake of medicine; Not pregnant; Not appearing to have an alcohol problem.

#### Procedures

Prior to inclusion and visit, the subjects were pre-screened per telephone, and study information was sent to possible participants. At the visit, the subject came fasting for the assessment by a rheumatologist. Demographics and clinical data were registered, joint scores were taken, and there was a possibility to ask questions about participation before giving written consent. If enrolled, fasting blood test was taken, and Patient-Reported General Health (PtGH) was assessed on a 100 mm Visual Analogue Scale (VAS) with 0 being best and 100 being worst case. Finally SF was aspirated from affected joint under ultrasound guidance. C-Reactive protein (CRP), anti-cyclic citrullinated peptide (anti-CCP) and rheumatoid factor (RF) were measured in the blood samples, and DAS28_CRP_ was finally calculated.

The HCs received written material prior to visit, where they were seen by a laboratory technician and had a possibility to ask questions concerning participation before giving written consent. Then demographics were registered and fasting blood tests were taken to confirm the health status as healthy.

#### Treatment of samples

From the blood samples cells isolated from EDTA-sampled full-blood were used directly for flow cytometry, and EDTA-plasma and serum was frozen at -80°C for further measurements.

Cells isolated in EDTA tubes from synovial fluid were also directly used for flow cytometry.

#### Protocol for flow cytometry of fresh blood and SF cells

Blood and SF cells were prepared and measured as described in S3 Text, particularly looking for neutrophils and monocytes and presence of C5aR.

#### Measurements of C5a and multiplex protein analysis

Plasma and SF C5a/C5a desArg were measured by a sandwich ELISA from BD Bioscience. In brief, microtiter plates were precoated with monoclonal antibody specific for human C5a/C5a desArg. A standard was supplied with the kit containing lyophilized human serum containing a specific amount of C5a desArg. A calibrator curve was prepared by spiking C5a desArg standard into standard diluent (animal serum), ranging from 0.078 ng/ml to 5 ng/ml. In addition, three quality control samples (QCs) were prepared covering Low (0.25 ng/ml), Medium (2 ng/ml) and High (4 ng/ml) level of the standard curve. Any C5a/C5a desArg in the samples was bound to the plate, and was detected by a mixture of biotinylated anti-human C5a antibody and streptavidin-HRP followed by colorimetric detection using Tetramethylbenzidine. The plate was read at 450 nm and reference at 620 nm on a Tecan Sunrise reader controlled by Magellan^TM^, Tecan Trading AG, Switzerland. The instrument response was exported to WatsonLIMS^TM^ software, ThermoFishes Scientific, where plasma concentrations were quantified.

SFs from RA patients were sent to Myriad RBM for analysis on their Human DiscoveryMAP® 175+ v1.0, a 45-biomarker Multi-Analyte Profile (MAP) designed to discern inflammatory biomarker patterns.

#### Migration assay in Boyden Chamber

Isolation of cells and the Boyden Chamber method to look at migration of granulocytes and monocytes are described in detail in S4 Text.

For all migration studies, only assays where migration towards SFs were significantly above migration towards medium, as determined by Student’s t-test, were included in the analysis.

### Statistics

Student’s t-test was used for group comparisons, Spearman’s Rank correlation test for correlations tests. Bonferoni correction was used to correct for multiple comparisons.

## Results

### Immunohistochemistry of synovium

**Fig 1:**
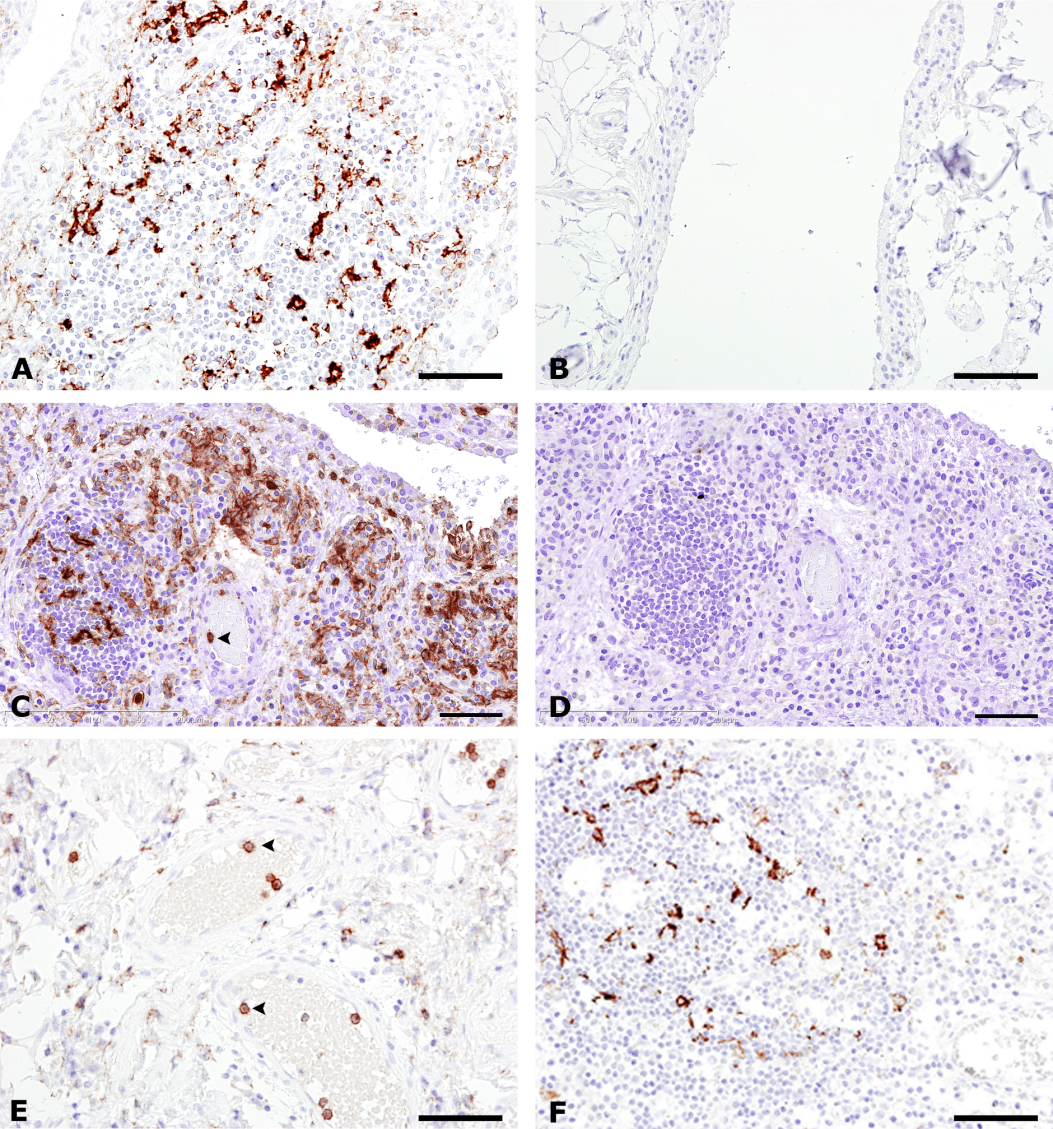
C5aR expression in the synovium from rheumatoid arthritis, osteoarthritis psoriatic arthritis, and non-inflammatory control. Immunohistochemical staining for C5aR (A, B, C, E and F), and for the IgG2a isotype control antibody (D). Synovium from patients with RA undergoing joint replacement is shown in (A), and synovectomy, (C, D). Synovium from patients with OA is shown in (E), and PsA in (F). Synovium from a non-inflammatory control is seen in (B). Numerous infiltrating C5aR^+^ cells (in brown) are seen in A, C and F. Intravascular C5aR^+^ neutrophils are seen in C and E (arrowheads). Note the lack of immunoreactivity in the non-inflammatory control (B). Nuclei (in blue) were counterstained with haematoxylin. Bars: (A, B, E and F) 50 μm, (C and D) 100 μm.

**Fig 2:**
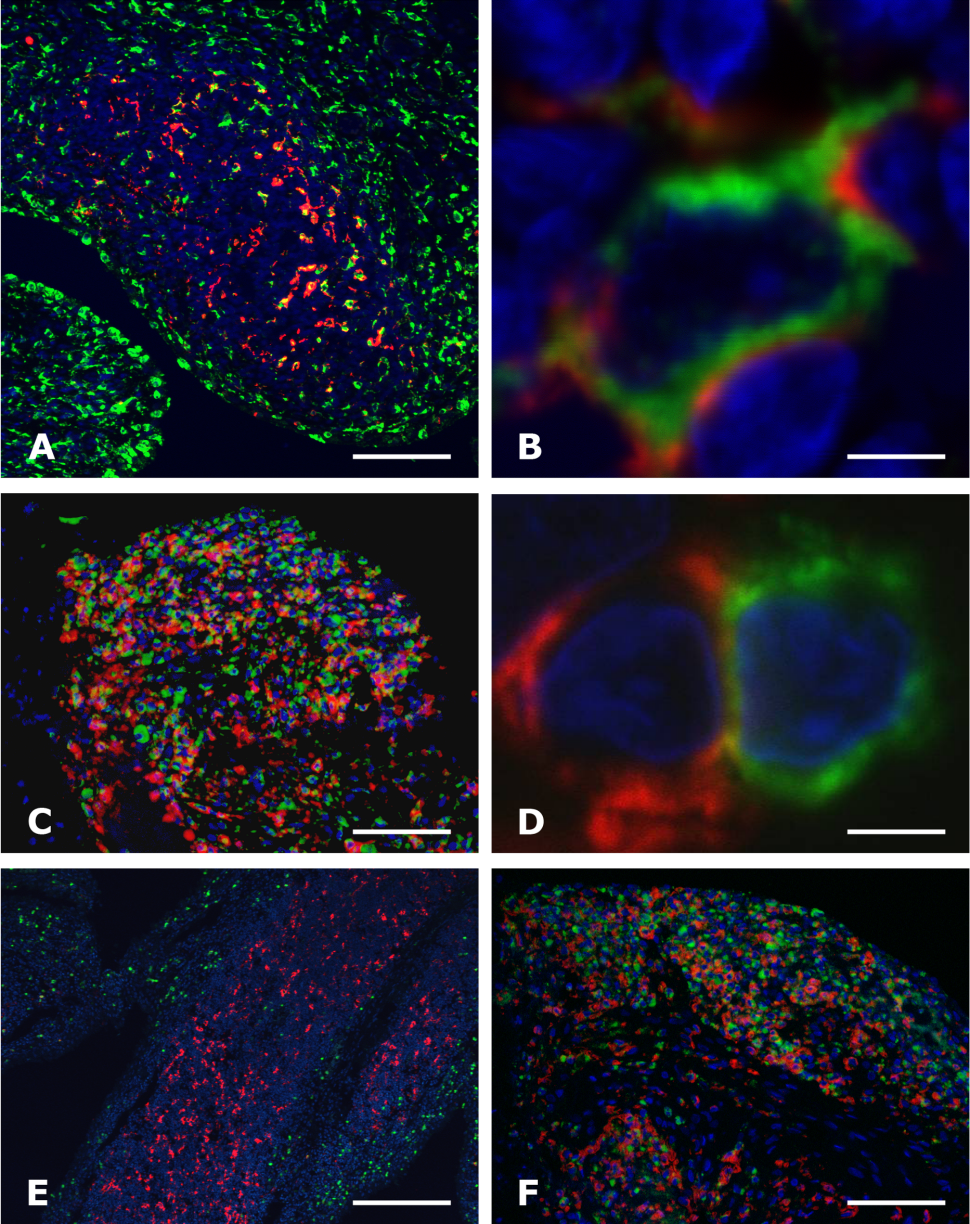
C5aR expression by macrophages and neutrophils, but not by T cells in the synovium of rheumatoid arthritis. Double immunofluorescence staining for C5aR (red signal A—F) and either CD68^+^ macrophages (green signal A, B and C), CD3^+^ T cells (green signal in D), or MPO^+^ neutrophils (green signal in E and F) of the synovium from rheumatoid arthritis patients undergoing joint replacement (A, B, D and E) or synovectomy (C and F), analysed by confocal microscopy (A, B, D and E) and epifluorescence microscopy (C and F). Framed area in E is seen in B. Note the co-localization of C5aR and CD68^+^ macrophages in A, B and C, and MPO^+^ neutrophils F, and lack of co-localization with a CD3^+^ T cell in D. The nuclei (in blue) were counterstained with Hoechst. Bars: (A) 100 μm, (B and D) 5 μm, (E) 500 μm, (C and F) 50 μm.

IHC analyses were performed on synovial tissue samples from joint replacement from patients with RA, PsA and OA, as well as on synovectomy biopsies from patients with RA. In synovial samples from joint replacement, infiltrating C5aR-positive (C5aR^+^) cells were demonstrated in 80% of patients with RA, in 100% of patients with PsA and in 73% of patients with OA, whereas no C5aR^+^ cells were infiltrating the synovium from non-inflammatory controls. C5aR^+^ cells were present in the synovial lining in all groups investigated. In synovial biopsies obtained by synovectomy, C5aR^+^ cells were present in 100% of patients with RA (Table 2, Fig 1). The C5aR^+^ infiltrating cells in OA synovial tissue were observed only when lymphoid aggregates were present, and with fewer positive cells present compared to RA. Double-immunostaining for CD68 and C5aR revealed that the majority of infiltrating C5aR^+^ cells in all patient groups was CD68^+^ macrophages (Table 2, Fig 2A–2C). The numbers referred to in Table 2 are given as positive samples out of the total number of samples, meaning a positive sample harbour C5aR^+^ cells and a negative sample do not. PMN/macrophage C5aR negative cells are present in the synovia, which could be explained by either a downregulation of C5aR in macrophages or lack of activation of macrophages leading to induction of C5aR expression.

**Table 2:** Summary of C5aR expression and characterization of C5aR-positive cells in the synovium from patients with RA, OA, PsA and from non-inflammatory controls.

Cell type	RA^1^ Joint replacement	RA^1^ Synovectomy	OA^1^	PsA^1^	Non-inflammatory control^1^^,^^2^
C5aR^+^ cells	28/35	8/8	11/15	10/10	3/7*
C5aR^+^MPO^+^ neutrophils	11/19	6/8	7/27	7/8	0/7
C5aR^+^CD68^+^ macrophages	11/16	7/7	10/21	7/7	3/4*
C5aR^+^CD3^+^ T cells	0/19	ND	0/27	0/2	0/5

^**1**^Number of positive samples/total number of samples included in the study.

^**2**^Histopathologically classified as being within normal limits.

Abbreviations: ND: Not done; OA: Osteoarthritis; PsA: Psoriatic arthritis; RA: Rheumatoid arthritis.

*Single or double-positive cells present in synovial lining.

For RA patients, the frequency of C5aR^+^ macrophages was slightly higher in biopsy samples compared to the findings in synovial samples from joint replacements. The C5aR^+^CD68^+^ macrophages were found in the synovial sublining layer interspersed in lymphoid aggregates and in the synovial stroma with up to 50% of the C5aR^+^ cells being CD68^+^ double-positive. All C5aR^+^ cells present in the synovial lining were found to be CD68^+^ macrophage-like synoviocytes. Germinal centres were not seen in the lymphoid aggregates. Double immunostainings for C5aR and CD3 (T cell marker), showed that in none of the diseased synovial samples (RA; OA and PsA) did CD3^+^ T cells express C5aR, but the C5aR^+^ cells were found in close proximity to the T cells (Table 2, Fig 2D). This observation might indicate the physical possibility of an indirect activation of T cells from C5aR^+^ cells.

Immunostaining for Myeloid peroxidase (MPO neutrophil marker) showed profound infiltration of neutrophils into the synovium from patients undergoing joint replacements in 58% of RA, in 88% of PsA, and 26% of OA patients, while no neutrophils were found in the synovium of non-inflammatory controls (Table 2). While 90% of neutrophils located in the lumen of synovial blood vessels were C5aR-positive, as determined by double staining for MPO and C5aR, only 5% of the neutrophils infiltrating the synovium from either RA or OA patients undergoing joint replacement were C5aR^+^ (Fig 2E). In contrast, up to 25% of the extravascular neutrophils in PsA and in RA samples obtained by synovial biopsies were double-positive for C5aR and MPO (Fig 2F). In all patient groups were the vast majority of intravascular MPO^+^ neutrophils found to be C5aR^+^.

### Flowcytometry of RA synovial cells

To assess the frequency of cell subsets of macrophages and infiltrating neutrophils present in RA synovial tissue, flowcytometry measurements of present neutrophils and macrophages were carried out on explants obtained from synovium from joints from nine RA patients were obtained. A detailed flowcytometry analysis of freshly isolated cells surface expression of C5aR confirmed the immunohistochemical findings described above. Infiltrating neutrophils and macrophages constituted 1.3% and 50%, respectively, of infiltrating CD45^+^ cells in the assessed synovia. The percentage of cell subsets varied between individuals. Within the viable hematopoietic cell compartment, CD14+ macrophages were most abundant, ranging from 23 to 70% of CD45+ cells, followed by T cells (6 to 30% of CD45+ cells).

### Flowcytometry of SF cells

Demographic data on participating patients is given in Table 3. In synovial fluids from RA and PsA patients, all neutrophils and monocytes expressed C5aR. Neutrophil and monocytes constituted on average 37% and 9% of the cells in the SF cells in RA, 58% and 7% in PsA patients. For comparison, these were 5% and 9% in OA, thereby questioning OA as a non-inflammatory control for the others.

**Table 3:** Demographic data on the subjects providing blood and synovial fluid.

	Male/ Female	Median Age (Range) Years Male/ Female	Disease Duration Months Male/ Female	±RF Male/ Female kIU/l	±anti-CCP Male/ Female U/l	CRP Male/ Female mg/l	DAS28_CRP_ Male/ Female
RA	4/25	62(52–76)/ 60(33–79)	85,5/ 121,1	+RF: 4/10 ÷RF: 0/15	+anti-CCP: 3/11 ÷anti-CCP: 1/14	7,25/ 15,6	3,91/ 3,78
PsA	4/7	36(36–40)/ 63(29–66)	177/ 69,4	+RF: 0/0 ÷RF: 4/7	+anti-CCP: 0/0 ÷anti-CCP: 4/7	69/ 12,3	4,40/ 3,42
OA	8/10	64(45–74)/ 72(56–85)	35,6/ 108,7	+RF: 1/0 ÷RF: 7/10	+anti-CCP: 0/0 ÷anti-CCP: 8/10	2,75/ 2,5	2,91/ 3,07
HC	8/24	52(32–72)/ 55(35–79	-	*+RF: 1/1 *÷RF: 7/22	+anti-CCP: 0/0 ÷anti-CCP: 8/24	1,13/ 1,30	-

+RF is defined as RF> 7 kIU/l

*One HC was not measured for RF. The two +RF were boarder-line +RF with 8 and 9 kIU/l. +anti-CCP is defined as <7 U/l. Disease duration is taken from date for diagnosis.

### C5a levels in SF

**Fig 3:**
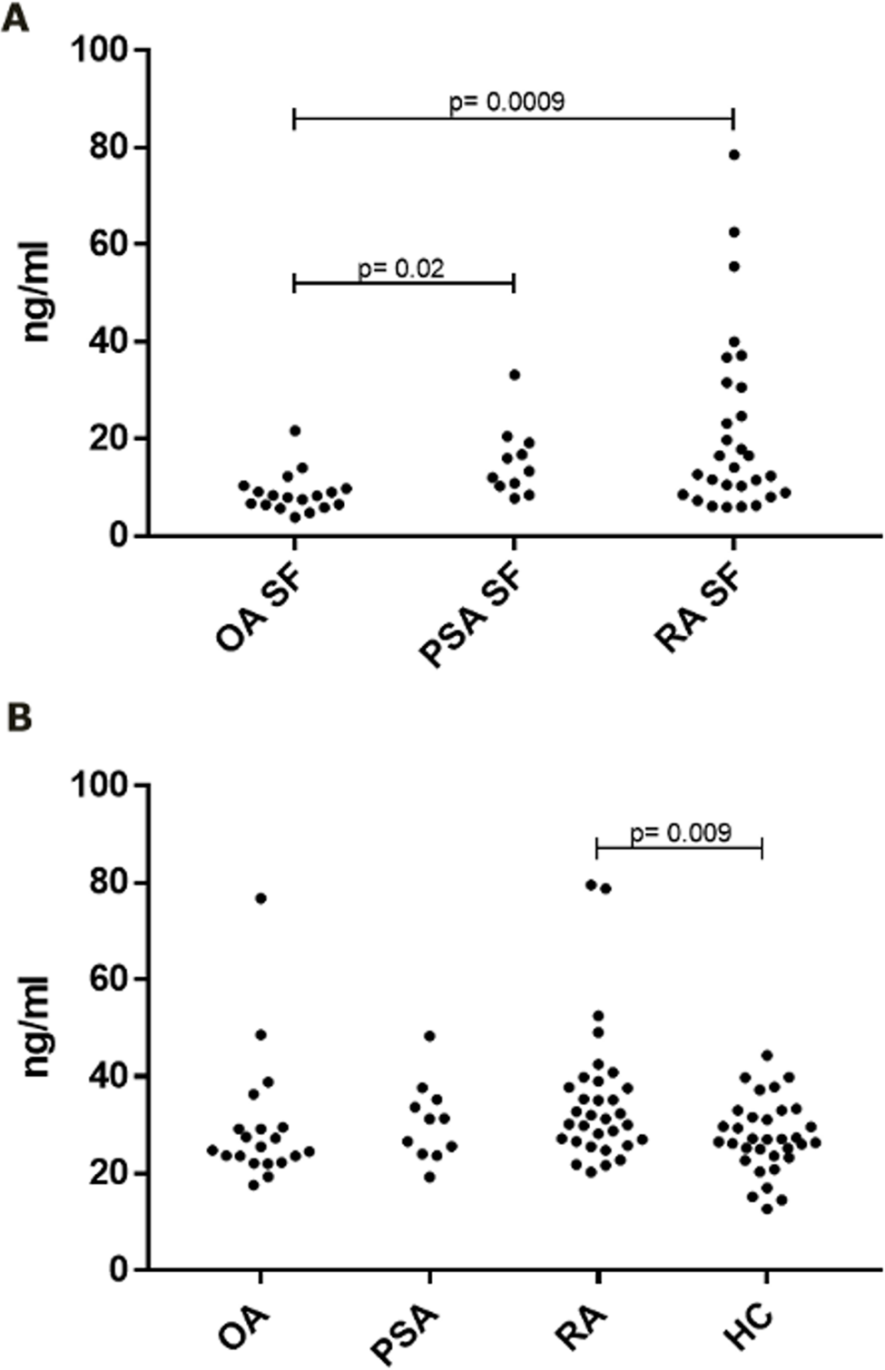
C5a levels in synovial fluids and plasma. A. Synovial fluid levels. B. Plasma levels. Statistical analysis was with the Student’s t-test. P-values are uncorrected for multiple testing.

The level of C5a in SF from RA and PsA patients was compared to the level found in OA patients. These were considered the best non-inflammatory controls available, although low inflammation is likely to be present due to the presence of a swollen knee and the above described findings of neutrophils and monocytes in SF. C5a levels in RA patients were found to be significantly elevated in RA compared to OA. The same was the case for PsA (Fig 3A).

In plasma the C5a level in RA was elevated relative to healthy controls (demographic data see Table 3), but not relative to OA (Fig 3B).

**Fig 4:**
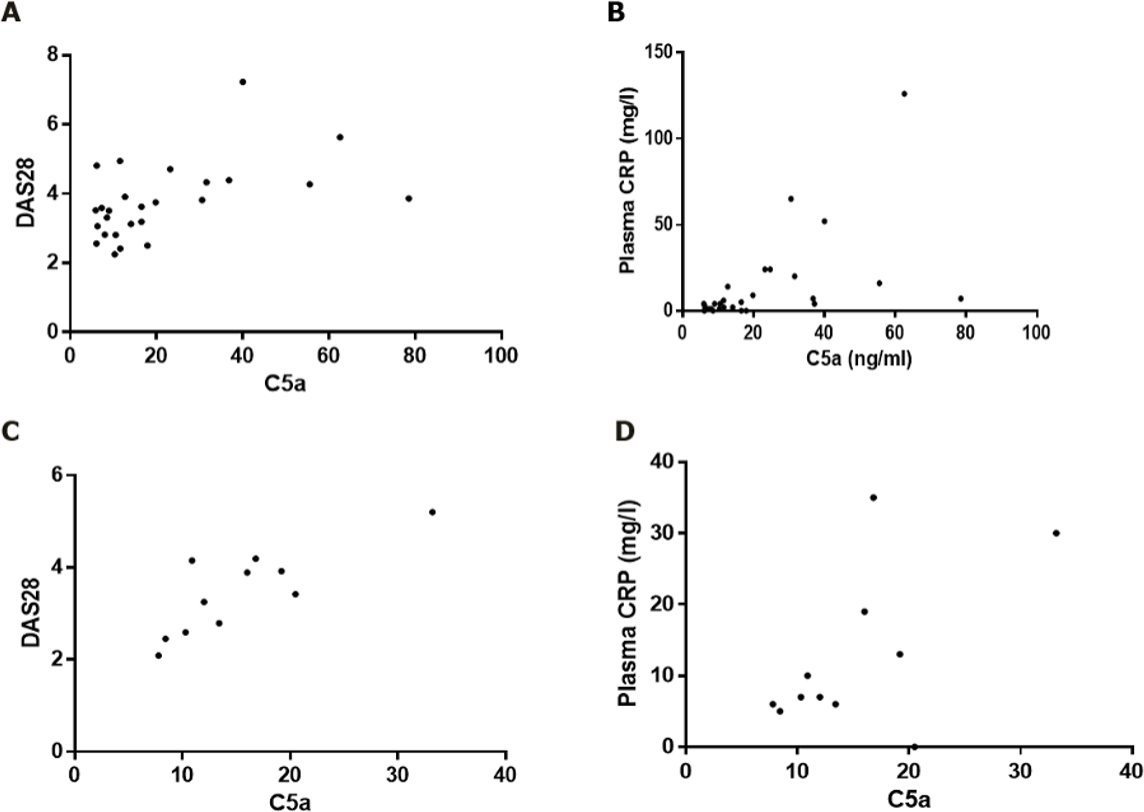
C5a levels in synovial fluids correlate to disease activity. A and B: From RA patients; synovial fluid C5a levels correlate to both DAS28 and plasma CRP (r values of respectively 0.5122 (p = 0.0075) and 0.6881 (p < 0.0001). C and D: From PsA patients; synovial fluid C5a levels correlate to DAS28 and plasma CRP (r value = 0.7727 (p = 0.0053), but not to CRP. Statistical analysis was with Spearman’s Rank correlation test.

In RA, SF C5a correlated both to DAS28 and to the plasma CRP level (p < 0.01 and p < 0.0001, respectively. For PsA, SF C5a correlated to DAS28 (p < 0.01) but not to plasma CRP, although a tendency was observed (Spearman’s Rank correlation coefficient = 0.4338), Fig 4.

Disease duration and levels of rheumatoid factor (RF) and/or anti-citrullinated protein antibodies (ACPAs) were not correlated to C5a levels. C5a plasma levels were in RA found to be correlated to the levels found in SF (Spearman’s Rank correlation coefficient = 0.4338; p = 0.0003), but not in PsA, although a tendency was observed (Spearman’s Rank correlation coefficient = 0.4273).

### Inflammatory markers in RA

RA SF samples were further subjected to a multiplex protein analysis and C5a levels were correlated to the measured inflammatory markers.

To correct for multiple comparisons, only markers where correlation to C5a is significant after Bonferoni correction of p-values were considered. A number of acute phase reactants (von Willebrand factor, Alpha-1-antitrypsin, CRP, Alpha-2-macroglobulin, haptoglobin and complement factor 3), a number of cytokines (IL-6, IL-10, IL-1RA, IL-1β, IFNγ) in addition to MMP9, soluble ICAM-1, soluble TNFR2, Factor VII and TIMP-1 were positively correlated to C5a, while MMP3 was negative correlated to C5a, Table 4.

**Table 4:** Biomarkers in RA SF correlating to C5a levels.

Marker	r-value	p-value
Von Willebrand factor	0.8403	< 0.0001
MMP9	0.8332	< 0.0001
IL-6	0.8134	< 0.0001
IL-10	0.8133	< 0.0001
IL-1RA	0.7354	< 0.0001
IL-1β	0.7302	< 0.0001
Alpha-1-Antitrypsin	0.7298	< 0.0001
ICAM-1	0.7297	< 0.0001
CRP	0.6951	< 0.0001
Alpha-2-Macroglobulin	0.6785	0.0001
Haptoglobin	0.6655	0.0002
Complement C3	0.6541	0.0002
TNFR2	0.6579	0.0003
IFNγ	0.6379	0.0003
Factor VII	0.6248	0.0005
TIMP-1	0.6235	0.0005
MMP3	-0.7994	< 0.0001

Statistics were carried out using the Spearman’s Rank correlation coefficient. Uncorrected p-values are given, but all the p-values are significant also after Bonferoni correction. Markers not fulfilling this criterion, but still positively correlated to C5a if no correction is performed, were CCL3, IL-8 and TNFα (all with p-values < 0.01), CCL4, VEGF, IL-4, IL12p40, IL-17A, IL-7, fibrinogen and soluble VCAM (all with p-values < 0.05). Stem cell factor was negatively correlated to C5a (p < 0.01).

### Immune cell migration

**Fig 5:**
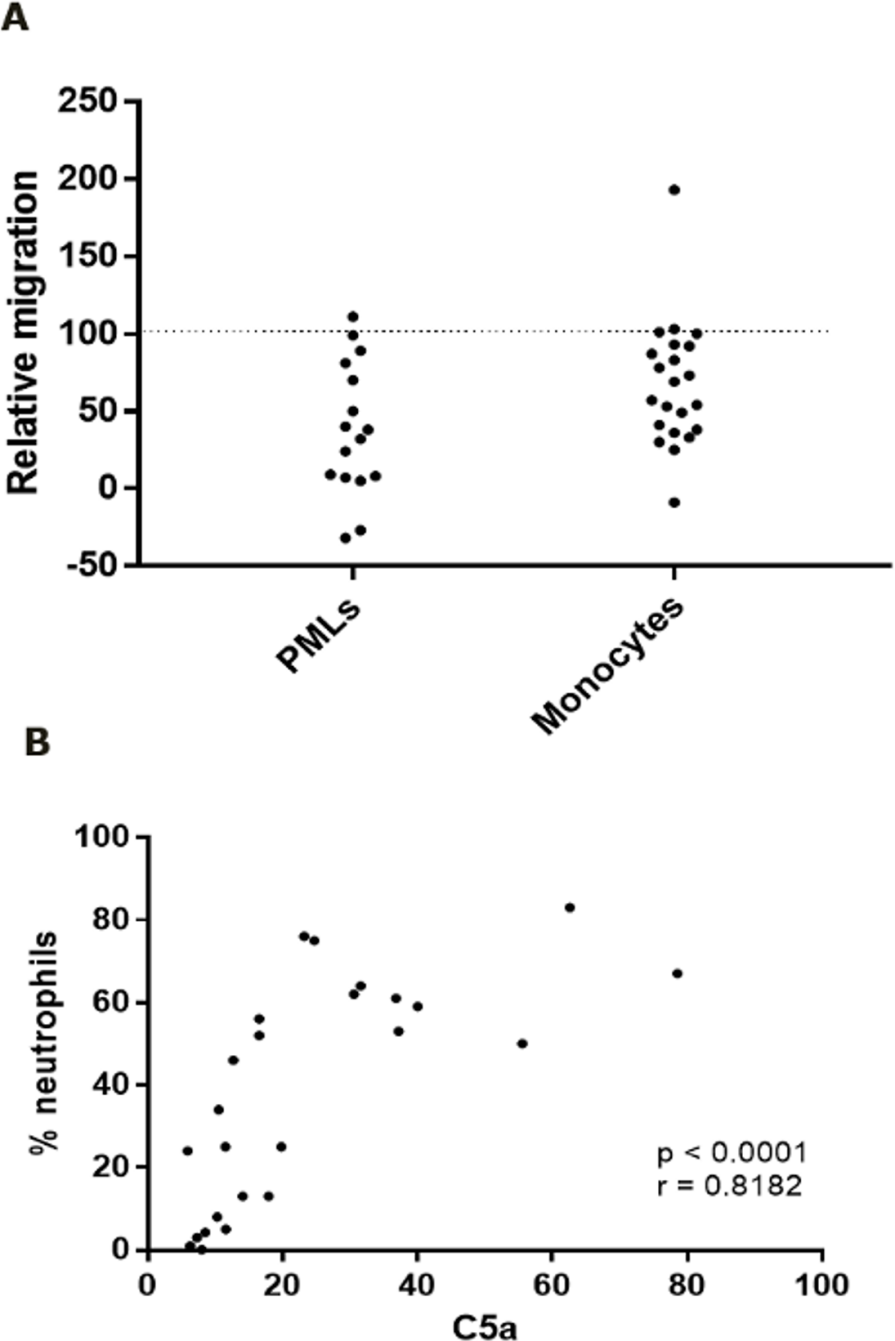
Anti-C5aR mAb inhibition of migration towards RA synovial fluids. A. PML and monocyte migration. All values were normalized by setting the migration with isotype control mAb added to 100. Average inhibition by anti-C5aR mAb was 60% (p = 0.014) for PMLs (n 0 16) and 33% (p = 0.0012) for monocytes (n = 22). B. Relative neutrophil number vs. C5a levels in RA SF. Percentage neutrophils in the synovial fluid correlate to synovial fluid C5a levels (r value = 0.8182; p < 0.0001). Statistical analyses were Student’s paired t-test in A and Spearman’s Rank correlation test in B.

To test the importance of C5a in chemo-attraction of granulocytes and monocytes to SFs from RA and PsA patients, the effect of a blocking anti-human C5aR mAb on leukocyte migration was determined using a Boyden chamber system. For RA SFs, granulocyte migration was significantly inhibited (p = 0.014) with an average inhibition of 60%, ranging from no inhibition to total inhibition, while monocyte migration was also significantly inhibited (p = 0.0012) with an average inhibition of 33%, ranging from no inhibition to total inhibition (Fig 5A). For a single subject, anti-C5aR treatment increased migration (corrected p-value < 0.05). We have no explanation for this. As it seemed that C5aR may have the largest effect on granulocyte migration into the joint, we also investigated if C5a levels in RA SF correlated to the percentage of neutrophils in SF. A significant correlation between levels of C5a and the relative proportion of neutrophils in SF from RA patients was indeed found (p < 0.0001; Spearman r = 0.082) (Fig 5B). Similar data were obtained when using the absolute number of neutrophils in the synovial fluids (p < 0.0001; Spearman r = 0.081). We only obtained data from 7 PsA SFs for granulocyte migration and from 2 PsA SFs for monocyte migration: Granulocyte migration was inhibited with an average inhibition of 79% (ranging from no inhibition to total inhibition), but this was not significant (p = 0.08). Monocyte migration inhibited 42% in one sample, but not at all in the other sample. There was no indication of a correlation of C5a levels to neutrophil proportion in the PsA SFs.

### C5a –C5aR correlation

Since C5a ligation leads to C5aR internalization, we investigated whether there was inverse correlation between C5a and C5aR on SF neutrophils and monocytes, as this would indicate that these cells had been subject to ligand binding. This was indeed the case: C5a exhibited inverse correlation to C5aR expression on both neutrophils (p = 0.0036 and Spearman’s Rank correlation coefficient = -0.5928) and monocytes (p = 0.0010 and Spearman’s Rank correlation coefficient = -0.6291) in SF from RA patients.

## Discussion

Our immunohistochemical investigations showed that C5aR-positive cells are infiltrating the synovial tissues of RA, PsA and OA patients, while no extravascular C5aR-positive cells are found in samples from non-inflammatory controls. Looking at RA, C5aR^+^ cells were found in 80% of the samples taken during joint replacement, and in 100% of samples obtained from synovectomy. The latter represents more active disease which is supported by the higher incidence of C5aR-positive cell. The two findings indicate an involvement of C5a/C5aR in the inflammatory process.

Presence of C5aR in inflamed synovial tissue from RA patients is reported before [51], but not further defined. We demonstrated that the majority of C5aR-positive cells in RA synovium, as well as in PsA and OA, were macrophages as determined by double-immunostaining for C5aR and CD68. We also observed that the C5aR^+^ cells were found in close proximity to the T cells. Although we have no data indicating any functional relevance of this observation, it is likely that the interaction plays a pathogenic role–either in contributing to local T-cell activation by antigen presentation by the myeloid cells to the T cell and/or to macrophage activation by the T cells. In this connection it must be noted that a direct role of the C5a-C5aR axis in T-cell activation by antigen-presenting cells have been observed, although this is disputed [11, 52, 53]. However, also C5aR-positive neutrophils were detected, albeit most infiltrating neutrophils were C5aR-negative. This may indicate that these neutrophils down-regulated C5aR after extravasation, since the vast majority of intravascular neutrophils were found to be C5aR-positive, as expected. Although the presence of neutrophils in the synovial fluid of RA patients is well-known [54], the detection of infiltrating neutrophils in the synovium is less recognized, despite of earlier reports [37, 40, 55]. These studies also report much fewer neutrophils present in synovium from OA patients–in agreement with our observations.

When measuring the level of C5a in SFs from RA patients, we confirmed increased C5a level in RA SF when compared to OA [12, 13]. We also showed C5a level to be correlated to DAS28. This is in agreement with earlier studies demonstrating that complement activation in SF of RA patients correlates to disease activity [5, 6], and specifically to DAS28 [7]. No correlation of C5a to RF was observed. This is in agreement with Doherty *et al* [5], but in opposition to Wouters *et al* [7]. However, in the latter study the level of RF correlates to DAS28. It could therefore be concluded that complement activation correlates to disease activity, but not to RF independently of this. The finding indicates that RF-containing immune complexes may not be driving complement activation in RA patients, in opposition to what has earlier been proposed [56]. We also did not see any correlation to ACPA titers. In summary, our results indicate that complement activation in patients with RA may not be due to activation of the classical pathway by immune complexes. Alternatively, it could happen via CRP, as suggested by others [57], since C5a levels correlate to CRP levels, both in SF and in plasma. Many other immunomodulatory markers correlated to local C5a titers. These included other acute phase reactants and the proinflammatory (IL-6, IL-1β and IFNγ) and anti-inflammatory cytokines (IL-1Ra and IL-10). This clearly indicates a correlation between local complement activation and acute inflammation. We observed a surprising inverse correlation between C5a and MMP3 in synovial fluids from RA patients. This is in opposition to other studies, where MMP3 correlates to other proinflammatory biomarkers [58], and we have no explanation for this apparent discrepancy.

To explore if C5a could be driving synovial influx in RA patients, we performed migration assays of neutrophils and monocyte towards synovial fluids from RA patients. Here we demonstrated that treatment with a blocking anti-C5aR mAb significantly reduced the migration of both cell types. This implies that pharmaceutical targeting of the C5a-C5aR axis may reduce synovial influx of both monocytes and neutrophils, as is indicated by earlier animal studies [15, 17, 19, 22]. A further indication of the importance of the C5a/C5aR in the described cell migration process is the finding that levels of C5a and the number of neutrophils in synovial fluid correlate.

In summary, we have provided data to support that the C5a-C5aR axis may be involved in the pathogenesis of RA. However, an oral C5aR antagonist, PMX53, was investigated in a small clinical trial of patients with RA, and that no effect on clinical score, macrophage infiltration or cytokine expression in synovia [59]. It should be noted that pharmacokinetic reporting in this study was limited to reporting of area under the curve, and that it is highly likely that the target was not inhibited at all times by the once daily dosings. Futhermore, it has later appeared that PMX53 is an agonist of Mas-related gene X2 (MrgX2) and via this receptor can induce mast cell degranulation [60, 61].The anti-C5 antibody, eculizumab, has also been reported to lack efficacy in patients with RA [1], although details has not been published. Again, it can be speculated that doses were too low, since the approved indications of eculizumab concerns C5-cleavage in the blood stream, so doses used there may not be sufficient for blocking C5-cleavage in a tissue, where an antibody will have limited access.

For PsA we obtained results similar to those obtained from RA patients: C5a titers were increased in synovial fluids, and titers correlated to DAS28 (albeit not to CRP levels). There was a tendency that anti-C5aR mAb inhibited neutrophil migration to PsA SFs, but this was not significant. When looking at OA, the SF showed invasion of immune cells, although not to the degree as seen in RA and PsA. Thus, OA cannot be considered a non-inflammatory control, but must be considered as a low-grade inflammation in the OA patients appearing with a swollen joint.

## Conclusion

The C5a-C5aR axis is important in the onset of inflammation and driving the influx of immune cells into the synovium in RA and PsA. The pathway may be a relevant target for treatment of these patients.

A larger study is though warranted to substantiate this in PsA.

OA is not a valid non-inflammatory model when comparing arthritic diseases due to a varying inflammatory profile.

## Supporting information

S1 Text Immunostaining for microscopy. (DOCX)

S2 Text Cell prepapration for flow cytometry from synovial tissue. (DOCX)

S3 Text Protocol for flow cytometry of fresh blood and SF cells. (DOCX)

S4 Text Setup for Boyden Chamber. (DOCX)

S1 Table Main data anonymised. (XLSX)

S2 Table Synovium FACS data anonymised. (XLSX)

S3 Table Boyden Chamber data anonymised. (XLSX)
