# Supplementary material for: C5a and C5aR are elevated in joints of rheumatoid and psoriatic arthritis patients, and C5aR blockade attenuates leukocyte migration to synovial fluid
Source: PLoS One. 2017 Dec 8;12(12):e0189017. doi: 10.1371/journal.pone.0189017 (PMC5722346; doi:10.1371/journal.pone.0189017)
Supplement: S1 Text — (DOCX) [file pone.0189017.s001.docx]

**S 1 Text**

**Immunostaining for microscopy**

The sections were deparaffinised in xylene and rehydrated in decreasing concentrations of alcohols. Antigen retrieval was performed in Tris-EGTA buffer (10mM; 0.5 mM), pH 9.0 in a microwave oven for 15 min. Endogenous peroxidase activity was blocked with 3% H_2_O_2_. Endogenous biotin was blocked by incubation with Avidin and Biotin blocking solutions for 10 min, according to the manufacturer. Non-specific binding was blocked by incubation with TBS containing 3% skimmed milk, 7% donkey serum, 3% human serum, and 3.2 mg/ml Poly-L-Lysine (PLL) for 30 min. The primary and secondary antibodies were diluted in a Tris buffer containing 0.5% skimmed milk, 7% donkey, 3% human sera, and incubation was performed overnight at 4°C (primary antibody), and 60 min at room temperature (secondary antibody). The first amplification step was performed by incubation with Vectastain ABC peroxidase kit, diluted in 0.1 M Tris-HCl buffer (pH 7,5) containing 0.5% Du Pont Blocking Reagent (TNB) for 30 min, followed by a second amplification step with incubation in biotinylated Tyramide for 6 min. The final amplification was performed by an additional incubation with the Vectastain ABC peroxidase kit, diluted as previously described for 30 min. The chromogenic reaction was achieved with diaminobenzidin. Nuclei were counterstained with haematoxylin and the sections were rehydrated, cleared in xylene and mounted with Eukitt. Evaluation of the slides was performed blinded to the observer.

***Double-immunofluorescence***

The immunohistochemical demonstration of the C5aR was performed as described above. Upon the second amplification with biotinylated Tyramide for 3 min were sections incubated with the Vectastain ABC peroxidase kit, diluted as previously described for 30 min. The final amplification was performed by incubation with Alexa-594 conjugated Tyramide diluted according to manufacturer for 6 min. Sections were then subjected to second rounds of microwave oven treatment in Tris-EGTA buffer (10mM; 0.5 mM), pH 9.0 for 10 min, blocking of endogenous peroxidase activity with 3% H2O2, and endogenous biotin by incubation with Avidin and Biotin blocking solutions for 10 min, respectively, according to the manufacturer. Additional blocking of non-specific binding was performed by incubation with TBS containing 3% skimmed milk, 7% donkey serum, and 3% human serum for 30 min. The second primary and secondary antibodies were diluted in a Tris buffer containing 0.5% skimmed milk, 7% donkey and 3% human sera, and incubation was performed overnight at 4°C, and 60 min at room temperature, respectively. The final amplifications were performed by an additional incubation with the Vectastain ABC peroxidase kit, diluted as previously described for 30 min, and incubation with Alexa-488 conjugated Tyramide. Nuclei were counterstained with Hoechst for 15 min, and the sections were mounted in fluorescence mounting media (Dako).

**Table A1** List of antibodies and conjugated detection kits used for immunohistochemical studies

| **Antibodies** | **Vendor** | **Cat. #** | **Clone** | **Iso-type** |
| --- | --- | --- | --- | --- |
| Primary |  |  |  |  |
| Mouse monoclonal anti-hC5aR | R&D System | MAB3648 | 347214 | IgG2a |
| Mouse monoclonal anti-hCD68 | Dako | M0814 | KP1 | IgG1 |
| Rabbit monoclonal anti-hCD3 | Neomarkers | RM-9107S1 | SP7 | IgG |
| Rabbit polyclonal anti-MPO | Osenses | DOM00001G | Polyclonal | IgG |
| Mouse isotype specific control | R&D System | MAB002 | 11711 | IgG1 |
| Mouse isotype specific control | Dako | X0943 | DAK-GO5 | IgG2a |
| Rabbit monoclonal isotype specific control | Cell Signalling Technology | 3900 | DA1E | IgG |
| Rabbit polyclonal control antibody | Jackson ImmunoResearch | 011-000-003 | Polyclonal | IgG |
| Secondary |  |  |  |  |
| Biotin conjugated Donkey anti-mouse | Jackson ImmunoResearch | 715-065-150 | Polyclonal | IgG |
| Biotin conjugated Donkey anti-rabbit | Jackson ImmunoResearch | 711-065-152 | Polyclonal | IgG |
| Detection systems |  |  |  |  |
| Peroxidase conjugated Avidin Biotin Complex (Vectastain) | Vector | PK-6100 | N/A | N/A |
| Biotin conjugated Tyramide | Perkin Elmer | NEL700 | N/A | N/A |
| Alexa-488 conjugated Tyramide | Invitrogen | T-20932 | N/A | N/A |
| Alexa-594 conjugated Tyramide | Invitrogen | T-20935 | N/A | N/A |

Abbreviations: MPO, Myeloperoxidase; N/A, not applicable.
