# Supplementary material for: C5a and C5aR are elevated in joints of rheumatoid and psoriatic arthritis patients, and C5aR blockade attenuates leukocyte migration to synovial fluid
Source: PLoS One. 2017 Dec 8;12(12):e0189017. doi: 10.1371/journal.pone.0189017 (PMC5722346; doi:10.1371/journal.pone.0189017)
Supplement: S2 Text — (DOCX) [file pone.0189017.s002.docx]

**S2 Text**

**Cell Preparation for flow cytometry from synovial tissue**

Single-cell suspensions of RA synovial tissue biopsies were prepared by mechanical disruption followed by enzymatic digestion for 1-2 h using Liberase TL (Roche, Welwyn Garden City, UK) and DNAse (Sigma-Aldrich Ltd, Dorset, UK). After passing the cells through a nylon mesh to exclude debris, cells were washed and resuspended in RPMI-1640 medium (containing 10% (v/v) FBS) at a density of 1x10^6^ cells/ml. For FACS analysis, dead cells were excluded with Live/Dead Fixable Aqua Dead Cell Stain (Life Technologies, Waltham, MA, USA). After gating on viable CD45+ cells within the monocytic gate, CD14 vs. CD15 staining was used to identify macrophages and infiltrating neutrophils. The following anti-human monoclonal antibodies from BD Biosciences (San Jose, CA, USA) were used: CD14-APC (clone M5E2), CD15-PECy7 (clone HI98), CD45-PerCP (clone TU116). Anti-human C5aR/CD88-PE (clone S5/1, Biolegend, London, UK) and mIgG2a-PE (clone MOPC-173, Biolegend, London, UK) were used to assess C5aR expression. To block non-specific Fc receptor-mediated antibody binding, FcR Blocking reagent (Miltenyi Biotec, Lund, Sweden) was used prior to antibody staining. Stained cells were fixed in Fixation Buffer (BD Biosciences, San Jose, CA, USA). Samples were acquired in a BD Fortessa flow cytometer (BD Biosciences, San Jose, CA, USA) and analyzed using FlowJo software (Tree Star, Ashland, OR, USA).
