# Supplementary material for: C5a and C5aR are elevated in joints of rheumatoid and psoriatic arthritis patients, and C5aR blockade attenuates leukocyte migration to synovial fluid
Source: PLoS One. 2017 Dec 8;12(12):e0189017. doi: 10.1371/journal.pone.0189017 (PMC5722346; doi:10.1371/journal.pone.0189017)
Supplement: S3 Text — (DOCX) [file pone.0189017.s003.docx]

**S3 Text**

**Protocol for flow cytometry of fresh blood and SF cells**

Blood cells were prepared by adding 100 µg/ml human IgG (Sigma I-4506, Sigma Aldrich, St. Louis, MO, USA) in staining buffer (PBS pH 7.40 with 0.2% human serum albumin (Sigma A-1653, Sigma Aldrich, St. Louis, MO, USA) and 0.09% w/v sodium azide) 1:10 by volume in staining tubes (Axygen MCT-150-L-C, Krackeler Scientific, Albany, NY, USA) to block non-specific Fc receptor-mediated antibody binding prior to start, and left after mixing for 15 minutes in the dark.

Isolated SF-cells, with a volume of approximately 500,000 cells, were treated in a similar way as the blood cells.

FACS-measurements were carried out on a BDFacsArray^TM^ WO114 (BD Bioscience, San Jose, CA, USA), which was adjusted with BD compensation beads prior to measurements.

Neutrophils were characterized by CD16 (CD16-APC-Cy7, BioLegend, San Diego, CA, USA) and monocytes were characterized by CD14 (CD14-PE-Cy7, BioLegend, San Diego, CA, USA). C5aR was determined with C5aR h7/16 antibodies (Novo Nordisk, Denmark). CD11b-positive (CD11b-APC-Cy7, BD Bioscience, San Jose, CA, USA) and CD62L-positive (CD62L-PE-Cy7, BioLegend, San Diego, CA, USA) cells were further localized to look at cell activity for these receptors. We also looked at CD203c-positve (CD203c-AP, Miltenyi Biotec Norden, Lund, Sweden) cells, but did not find any.

Data were analysed using the BDFacsArray^TM^ WO114 (BD Bioscience, San Jose, CA, USA) software.
