# Supplementary material for: C5a and C5aR are elevated in joints of rheumatoid and psoriatic arthritis patients, and C5aR blockade attenuates leukocyte migration to synovial fluid
Source: PLoS One. 2017 Dec 8;12(12):e0189017. doi: 10.1371/journal.pone.0189017 (PMC5722346; doi:10.1371/journal.pone.0189017)
Supplement: S4 Text — (DOCX) [file pone.0189017.s004.docx]

**S4 Text**

**Setup for Boyden Chamber**

***Isolation of granulocytes for the Boyden Chamber Study***

Human granulocytes (PolyMorphoNuclear leukocytes (PMN); granulocytes)

were obtained from blood drawn into vials containing EDTA, from humans from the voluntary and consenting donor corps of Novo Nordisk A/S in Måløv, Denmark. The blood cells were separated by centrifugation of blood (4 parts) through a Ficoll-Paque PLUS (GE Health Care) gradient (3 parts) for 30 min (400 x g) at room temperature. The PMN-containing layer was suspended in PBS (phosphate buffered saline) containing dextran-500 (Sigma) for 1 h to remove contaminating erythrocytes. The supernatant was centrifuged for 5 min (250 x g) at room temperature and remaining erythrocytes was osmotically lysed using 0.2% NaCl for 55 s. The solution was made isotonic by 1.2 % NaCl + PBS and centrifuged at 250 x g for 5 min, before the osmotic lyses was repeated. After centrifugation the PMNs was resuspended in reaction mixture (RM): HBSS (cat no 14175 Gibco) contained NaCl 137mM, KCl 5.3mM, Na2HPO4 0.33mM, NaHCO3 4mM, KH2PO4 0.44mM, Glucose 5mM; supplemented with MgSO4 ,7H2O, 0.4mM, MgCl2, 0.5mM, CaCl2 0.5mM, HEPES 20mM. Cell density was determined by NucleoCounter (Chemometec). The PMN suspension contained >95 % neutrophils as evaluated by microscopy of Giemsa-stained samples. The cells were loaded with the fluorescent dye Calcein, AM, (Fluka). Calcein was dissolved in DMSO and diluted 1000X in RM with cells (2 mill cells per ml) to yield a concentration of 10 µM. The suspension was incubated for 30 min in incubator at 37°C and then washed 3 times with RM to remove excess Calcein. Finally the cells were resuspended in RM (4 x10^6^ cells/ml).

***Chemotactic migration of PMNs***

The migration of PMNs was evaluated by the Boyden chamber technique using FluoroBlok® 3μm pore size 96- well (cat. No. 351161.BD Falcon (VWR)). The upper chamber i.e. the inserts containing Fluoroblok membrane was coated with human fibrinogen (cat noF3879-1G, Sigma) in 1mg/ml PBS at 37°C for 2 hrs. After washing the membranes were blocked with a solution containing 2% bovine serum albumin (BSA) in PBS. After another wash using RM, 105 Calcein-loaded PMNs in 25 μl RM and 25 μl of test compound at 100 μg/ml in RM (either antibody directed against the C5a-receptor, anti-C5aR h7/16 (0151-0000-0000 2A, CoV 19 apr.2011, IgG4) or a control antibody (HzANTP (OP004 14-07-2009, IgG4) were added to each well and placed in an empty receiver plate The lower chamber/receiver plate contained 225 µl of the control solution (RM), the chemoattractant hC5a (Sigma, C5788) at 10 nM in RM or the synioval fluid (SF) sample from the patients. The SF sample was diluted 2x using RM containing 0.2 % EDTA. The latter was added to avoid the formation of C5a and other complement factors from the SF during the incubation at 37°C. Groups using hC5a or control solution comprised of 5- 6 wells whereas groups using synovial fluid comprised of 2 x 4 wells – one group with the control antibody HzANTP and one group with anti-C5aR h7/16. Migration was started by inserting the upper plate with cells in the pre-filled receiver plate. The plate assembly was measured at 485/538nm, 37°C every 5 min for 60 min in a plate reader (SpectraMax, Molecular devices, or Fluoroscan, Thermo Labsystems.). The peak value was usually attained after 30 min and the number relative fluorescence units were used as a measure of migration. The wells are read from the bottom. The FluoroBlok™ membranes block the passage of light with a wavelength of 490-700 nm and thereby prevent detection of the labelled cells in the upper chamber. Only when the cells have migrated through the membrane to the lower chamber they are detected.

***Isolation of human monocytes for migration studies***

Buffy coats from healthy individuals were obtained anonymously from the Blood Bank, Copenhagen University Hospital (Rigshospitalet). All donors gave informed consent according to the protocol approved by The Ethics Committee for Copenhagen, Denmark, for research use (approval #H-D-2008-113). Peripheral blood mononucleated cells were isolated from the Buffy coats using pre-warmed Histopaque (cat.no. 1077, Sigma,St, Louis,USA) in 50ml LeucoSep tubes. Purification of the human monocytes was done using Human Monocyte enrichment cocktail (Rosette sep, Stemcell cat no 15068, Grenoble, France). The monocytes were stained using 10 µM calcein AM (cat no.17783, Fluka, St, Louis, USA) in Reaction Mixture (RM), (HBSS cat no 14175 Gibco, Paisly, UK + MgSO4, 0.4 mM, MgCl2, 0.5 nM , CaCl2 ,0.5 nM , HEPES, 20mM, pH 7,4) in the incubator for 30 min. After 3 times wash with RM and centrifugation (250x g for 5 min) the cells were re-suspended in RM 2.5 mill cells/ml and were ready for migration.

**S*etup for chemotactic migration of human monocytes***

Falcon™ 96-Multiwell FluoroBlok™ Cell Culture Inserts was used as described above for PMNs except for the coating of the FluoroBlok membrane. Before migration the membranes were coated with human fibronectin (Sigma F0895-2mg) (40 µg/ml in sterile water) using 50uL/insert at 37°C for 2 hrs. After washing twice with 50μL sterile water the membranes were blocked for 30 min with PBS + 2% BSA, 50uL/insert at room temperature and finally washed twice with 50 μl RM.

We compared the effect of hC5a at 10 nM with the well-known monocyte chemoattractant monocyte chemotactic protein 1 (MCP-1) (rhCCL2/MCP-1 R&D systems, cat no 279-MC/CF) and found that they were equipotent. Therefore we only used C5a. Calcein-loaded monocytes 105, were added to each well and placed in the receiver plate (lower chamber) which contained 225 µl of the control solution (RM), the chemoattractant hC5a (Sigma, C5788) at 10 nM in RM or the synioval fluid (SF) sample from the patients prepared as described above. Again groups employing hC5a comprised of 5- 6 wells whereas groups using synovial fluid comprised of 2 x 4 wells comprised of 2 x 4 wells **–** one group with the control antibody HzANTP and one group with anti-C5aR h7/16. The whole plate was measured every 5 min for 2 h at 485/538 nm and 37°C using the methods described above. We employed a longer migration time since monocytes migrate slower than PMN.
